# Overexpression of salicylic acid methyltransferase reduces salicylic acid-mediated pathogen resistance in poplar

**DOI:** 10.3389/fpls.2022.973305

**Published:** 2022-10-31

**Authors:** Huixia Dong, Wei Zhang, Yongxia Li, Yuqian Feng, Xuan Wang, Zhenkai Liu, Dongzhen Li, Xiaojian Wen, Shuai Ma, Xingyao Zhang

**Affiliations:** ^1^ Key Laboratory of Forest Protection of National Forestry and Grassland Administration, Ecology and Nature Conservation Institute, Chinese Academy of Forestry, Beijing, China; ^2^ College of Life Science, Henan Normal University, Xinxiang, China; ^3^ Co-Innovation Center for Sustainable Forestry in Southern China, Nanjing Forestry University, Nanjing, China; ^4^ Resources Management, Chinese Academy of Forestry, Beijing, China

**Keywords:** poplar, salicylic acid methyltransferase, salicylic acid, methyl salicylate, pathogen resistance

## Abstract

Salicylic acid (SA) is generally considered to be a critical signal transduction factor in plant defenses against pathogens. It could be converted to methyl salicylate (MeSA) for remote signals by salicylic acid methyltransferase (SAMT) and converted back to SA by SA-binding protein 2 (SABP2). In order to verify the function of SAMT in poplar plants, we isolated the full-length cDNA sequence of *PagSAMT* from 84K poplar and cultivated *PagSAMT* overexpression lines (OE-2 isolate) to test its role in SA-mediated defenses against the virulent fungal pathogen *Botryosphaeria dothidea*. Our results showed that after inoculation with *B. dothidea*, OE-2 significantly increased MeSA content and reduced SA content which is associated with increased expression of *SAMT* in both infected and uninfected leaves, when compared against the wild type (WT). Additionally, *SAMT* overexpression plant lines (OE-2) exhibited higher expression of pathogenesis-related genes *PR-1* and *PR*-5, but were still susceptible to *B. dothidea* suggesting that in poplar SA might be responsible for resistance against this pathogen. This study expands the current understanding of joint regulation of SAMT and SABP2 and the balance between SA and MeSA in poplar responses to pathogen invasion.

## Introduction

Poplars are the primary tree used for timber production in the northern hemisphere and the area of planted poplars is increasing rapidly, particularly in China, South Korea, and the United States (Heilman, 1999). China currently has with the largest area of poplar plantations, with about 8 million hectares (Zeng, 2002). The 84K poplar cultivar is a desirable clone that was bred produced through hybridization between *Populus alba* and *P. glandulosa* parents. It was introduced into China from Korea in 1984 by the Chinese Academy of Forestry. 84K poplar, also known as green poplar, are tall trees that produce quality material and do not produce flying catkins in the spring. In 2000, it was deemed the ideal tree species for landscape cultivation in northern China, and was also the preferred species for construction of the Three-North Forest Shelterbelt Program, plain greening, garden greening, and green channel construction (Zhou et al., 2007). However, the rapid occurrence of poplar diseases and insect pests has caused great losses in China. Poplar canker, caused by the fungal pathogen *Botryosphaeria dothidea* is one of the most devastating diseases of poplar. In recent years, this pathogen’s geographical distribution and host range have continuously expanded to 16 provinces in China, and can infect over 100 species of poplar (Zeng, 2002; Liao et al., 2014). Studies on interactions between poplar and *B. dothidea* range from focusing physiology, biochemistry (Hu and Zhu, 1997), and histopathology (Liang et al., 2005) to the molecular biology of genes mechanistically underlying pathogenesis (Morse et al., 2007; Li et al., 2018).

After inoculation with *B. dothidea*, salicylic acid (SA) (Dempsey et al., 1999; Dong, 2001) and pathogenesis-related genes (PRs) (Uknes et al., 1992; Ryals et al., 1996) in plants have been found to be closely related with disease resistance. SA could be converted into methyl salicylate (MeSA) by salicylic acid methyltransferase (SAMT) (Ross et al., 1999; Fukami et al., 2002), and some MeSA can be volatilized into the air (Tieman et al., 2010). SA generated at the inoculation site cannot be moved to other place of plant body (Vernooij et al., 1994), however, since MeSA can be volatilized it can be translocated non-inoculated sites. At the non-inoculated site, SABP2 can convert MeSA back into SA (Du and Klessig, 1997; Kumar and Klessig, 2003; Park et al., 2007; Vlot et al., 2008; Li et al., 2018), thus enhancing the ability of plants to resist re-infection of still healthy plant parts. That is, SAMT and SABP2 proteins play important roles in the SA pathway of plants. Previous studies have found that *OSBSMT1*, a salicylic acid/benzoic acidcarboxyl methyltransferase gene from *Oryza sativa*, when transformed into *Arabidopsis thaliana*, induced considerably higher levels of MeSA compared to the wild-type, some of which was vaporized into the surroundings. When this overexpressed transgenic plant was infected with the bacterial pathogen *Pseudomonas syringae* or fungal pathogen *Golovinomyces orontii*, SA did not accumulate and the plants were more susceptible to disease than the wild-type, indicating SA could be involved in the resistance response to these pathogens. In plants, downstream signal transduction of the initial immune response stimulated SA production, resulting in the induction of chemicals like flavan-3-ol, chlorogenic acid, gluconic acids and a large number of defense genes, especially PRs (Moore et al., 2011; Xue et al., 2013; Ullah et al., 2019). The expression patterns of PR-1 and PR-5 were considered to be markers for SA-dependent systemic acquired resistance (SAR) (Molinari et al., 2014; Li et al., 2018). However, the *PR-1* gene, used as a marker for SA responses, was minimally induced in the SA-hyperaccumulating transgenic plant line (Molinari et al., 2014; Ullah et al., 2022). In poplar, the relationship between SA and PRs in transgenic lines remains unclear.

We hypothesized that SAMT of 84k poplar (*PagSAMT*) regulates SA and MeSA contents in woody plants, thus playing a role in plant pathogen resistance. Here, we studied the function of *PagSAMT* in detail, and the possible molecular mechanism responsible for disease resistance in 84K poplar by analyzing the synthesis of SA and MeSA and the expression of *PagSAMT* and PR genes in the signaling pathway.

## Methods

### Production of transgenic plants

Poplar 84K cultivar (*Populus alba × Populus glandulosa*) was acquired from State Key Laboratory of Tree Genetics and Breeding, Chinese Academy of Forestry. The full-length open reading frame of poplar 84K SAMT gene, *PagSAMT*, (submitted to NCBI, accession Number: KT429670), was amplified and cloned into the pDNOR222.1 entry vector (Life technologies, Carlsbad, California, U.S.) using the Gateway cloning system (Invitrogen) with primers of attB-SAMT-GBD-F: GGGGACAACTTTGTACAAAAAAGTTGGAatggaggttgctcaagtgcttc and attB-SAMT-GBD-R: GGCGGCCGCACAACTTTGTACAAGAAAGTTGGGTAcatccctttctagtcacggaaac. The entry clones were then subcloned into the pMDC32 expression vector to produce the 35S::*PagSAMT* construct. Then *PagSAMT*-overexpressing transgenic popular 84K plants, designated OE-*PagSAMT*, were obtained by Agrobacterium-mediated transformation, using hygromycin resistance for selection (Koo et al., 2007).

### Multiple sequence alignment and phylogenetic tree construction

Multiple sequence alignment was performed on selected known SAMT proteins sequences and that of *PagSAMT* using Mega and GeneDoc program. The phylogenetic tree was constructed with PAUP3.0 software using the maximum likelihood method.

### Plant materials and pathogen infection

In our study, the *B. dothidea* isolate used (deposited in the forest pathology laboratory of the Research Institute of Forest Ecology, Environment and Protection, Beijing, China), was isolated from poplar bark exhibiting canker symptoms from plants grown in the Haidian District of Beijing, China. Before inoculation, *B. dothidea* was cultured on 2% potato dextrose agar (20 g potato extract, 20 g dextrose, 17 g agar, 1 L water) for six days at 25°C in the dark. Then hyphae were picked and inoculated into potato dextrose (20 g potato extract, 20 g dextrose, 1 L water) liquid culture medium for three days at 25°C, with shaking at 200 rpm. The fungal mycelium was collected by filtration, excess moisture was removed with blotting paper, and then put into 1.5 mL centrifuge tubes. The hyphae were frozen in liquid nitrogen and ground as finely as possible. Approximately 1 g of frozen *B. dothidea* powder was weighed into test tubes containing 150 mL of PDA. The samples were pre-warmed at 25°C for six hours, with shaking at 200 rpm before inoculation.

When tissue cultured plantlets of wild-type and transgenic OE-*PagSAMT* poplar reached 10 cm in the culture medium, they were moved to the greenhouse at the Chinese Academy of Forestry (CAF, Beijing, China). Plants were grown in soil at 25°C under a 12:12 h light/dark cycle. Eighteen plantlets of OE-*PagSAMT* or wild poplar plants, all approximately 40 cm tall, were selected for inoculation.

*B. dothidea* inoculations were performed on leaves 5 and 6 from the top leaf of wild-type and transgenic OE-*PagSAMT* poplars at the same time. Wounds were made using dissecting needles, at a spacing of 1.5 mm. Wounded leaves were dipped in mycelium suspension, and the plants were cultured in a climate chamber maintained at 25°C, 85% relative humidity (RH), under a 12/12 h light/dark cycle (Tieman et al., 2010). At times 0 (three healthy plants), 1, 2, 3, 4, and 5 days after *B. dothidea* inoculations, the leaves of three plantlets (inoculated or non-inoculated on the upper part of inoculated ones) were cut to < 5 mm^2^ separately and stored in liquid nitrogen. The leaves of each plantlet were equally separated into 3 weighted parts for the determination of SA and MeSA, and RNA extraction (n = 3).

### RNA Isolation and RT-qPCR

Total RNA was extracted using the TianGen RNAprep Pure Plant Kit (Polysaccharides and Polyphenolics-rich) (TianGen, Beijing, China). First-strand cDNA synthesis was carried out with approximately 1.0 μg RNA using the FastQuant RT Kit (with gDNase) (TianGen, Beijing, China). Specific RT-PCR primers were designed to produce amplicon lengths of 150–250 bp and melting temperatures of 58-60°C using Primer3 software (http://www.simgene.com/Primer3). The amplified fragments were separated by agarose gel electrophoresis. Reverse transcription quantitative PCR (RT-qPCR) was performed in quadruplicate using the KAPA SYBR^®^ FAST qPCR Kit Master Mix (2X) Universal (KAPA Boston, Massachusetts, United States) on a Roche LightCycler 480 (Roche Applied Science, Germany) according to the instrument guidelines. To identify the specificity of each primer pair, melting curve analysis was performed according to the manufacturer’s instructions. Expression was normalized relative to the control gene (β-tubulin) using Roche LightCycler advanced relative quantification analysis. Primers used for RT-qPCR were as follows: SAMT forward, CGGAGTCCCTGGTTCTTTCT, SAMT reverse: CTCCTCCGAGCGACACTTTA, SABP2 forward: GGTGCTGGCAGAAGTTCAAA, SABP2 reverse: CTCCAAGGCTGTGTCCTACT, PR-1 forward: GCTATAACAATCCCTCTATCCCTT, PR-1 reverse: CCACACAATATTTCCAACACCTAC, β-Tubulin forward: GCACCAACTTGTTGAGAATGC, β-Tubulin reverse: TTTCAACTGACCAGGGAACC.

### Measurement of SA content

SA was measured as previously described (Verberne et al., 2002; Zhang et al., 2009; Li et al., 2018). Approximately 150 mg of leaf tissue samples were put into 1.5 mL centrifuge tube, frozen in liquid nitrogen and ground into a fine powder, then extracted with 90% and 100% methanol. The extracts were blow-dried using nitrogen, sequentially put into 0.25 mL trichloroacetic acid (5%) and shaken for 2 min, then 0.8 mL of ethyl acetate and cyclohexane mixture (1:1, V/V) were added to the extract. The top organic layer was transferred to a new centrifuge tube and blow-dried by nitrogen. The pellets were dissolved in 1 ml of methanol used for SA. SA analysis was performed on an HPLC (Waters 2695, America) column, Thermo ODS HYPERSIL (4.6 mm×200 mm, 0.5 μm). The eluent was 52% methanol, pH 3, at a flow-rate of 0.80 mL/min, retention time of 10.943 min, and injection volume of 20 μL. Standard substance SA were diluted to concentration of 10^-3^, 10^-4^ 10^-5^, 10^-6^, 10-7 μg/μL with methanol, and the concentration of the SA components were calculated using a standard curve. The analysis was repeated with three times for each sample.

### Measurement of MeSA content

MeSA was extracted from tissues with methanol and n-hexane (Wen et al., 2021). 1g of leaf tissue was frozen in liquid nitrogen, ground into a fine powder, then extracted twice with 1ml of methanol. 1ml n-hexane was then added and the samples were mixed and layered. The top n-hexane layer was used for MeSA measurement by gas chromatography (GC) equipped with the ABEL-BONDED AB-FFAP capillary column. The initial oven temperature was held at 50 °C for 1 min and was programmed to increase at 10°C per min to 200°C for another 30 min. The injection temperature was 250°C and the detector temperature was 270°C. Pressure was 5 PSI. Standard substance MeSA was diluted to concentrations of 10^-3^, 10^-4^ 10^-5^, 10^-6^, 10-7 μg/μL with n-hexane, and the concentration of the MeSA components were calculated using a standard curve. The analysis was repeated with three times for each sample.

To measure the vaporized MeSA, inoculated plants were placed in a collection bag (35×43 cm, Toppits, Sweden) for the duration of the experiment (5 days), letting the plant grow naturally. Two silicone tubes were put into the entrance of the bag. The outer end of one of the tubes was linked with an activated charcoal filter. The other was connected to an adsorption column (Camsco, USA) packed with 200 mg of Porapak Q adsorbent (mesh80-100, Supelco, Bellefonte, PA). This Porapak Q column, which was replaced every 30 min, trapped MeSA and other volatile matter. The other ends of the filter and adsorption columns were linked with the outlet and inlet of the atmospheric sampling instrument, respectively. The air in the bag was vacuumed and filled with clean air through the activated charcoal filter. After inoculation, the volatile compounds were collected into adsorbent traps and air was pumped at 200 mL/min from the outlet. Trapped volatiles were eluted with n-hexane and analyzed by GC.

### Data analysis

The differences of *PagSAMT* gene expression levels between different transgenic lines were analyzed by one-way ANOVA with Tukey’s test. The SA and MeSA concentration and *PR* genes, *SAMT* and *SABP2* expression differences across the time series of a certain line were analyzed by one-way ANOVA with Tukey’s test and between 84K and OE-2 lines were analyzed by unpaired two-tailed Student’s t tests using SPSS 18.0 software (Green and Salkind, 2010).

## Result

### Cloning and sequence analysis of *PagSAMT*

**Figure 1:**
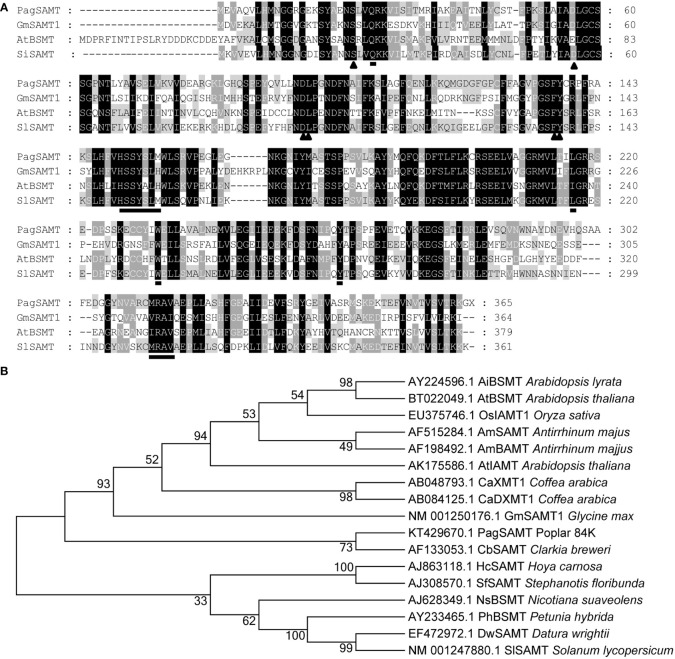
Sequence alignment of *PagSAMT* with other plant methyltransferases. **(A)** Multiple sequence alignment of *PagSAMT* with selected known SAMTs. The triangles indicate S-adenosyl-L-methionine (SAM) binding residue. The underlined amino acids represent SA binding residues. GmSAMT1, *Glycine max* SAMT1 (NM_001250176.1); AtBSMT, *Arabidopsis thaliana* BSMT (BT022049.1); SISAMT, *Solanum lycopersicum* SAMT (NM_001247880.1). **(B)** Phylogenetic tree containing *PagSAMT* and other members of the plant SABATH family of methyltransferases. The numbers at nodes in the phylogenetic tree represent the bootstrap values for 1000 replicates.

The full-length open reading frame of the *PagSAMT* gene was cloned and sequenced. The protein encoded sequence by this gene was also predicted. The translated protein sequence of the poplar *PagSAMT* gene was aligned with previously reported SAMTs from other plant species (**Figure 1A**). Among characterized SAMTs, PagSAMT had the highest sequence similarity to the SAMT from *Solanum lycopersicum* (*SISAMT*), sharing 56% identity. Most SAM-binding and SA binding domains are conserved among the aligned proteins.

PagSAMT belong to the plant SABATH family of methyltransferases, which include other methyltransferases, such as benzoic acid carboxyl methyltransferase (BAMT) (Murfitt et al., 2000), jasmonic acid methyltransferase (JAMT) (Seo et al., 2001) and indole-indole-3-acetic acid methyltransferase (IAMT) (Qin et al., 2005). Phylogenetic analysis showed that PagSAMT is more closely related to the known SAMTs than the other types of methyltransferases (**Figure 1B**).

### Production of transgenic *PagSAMT* overexpressing lines

**Figure 2:**
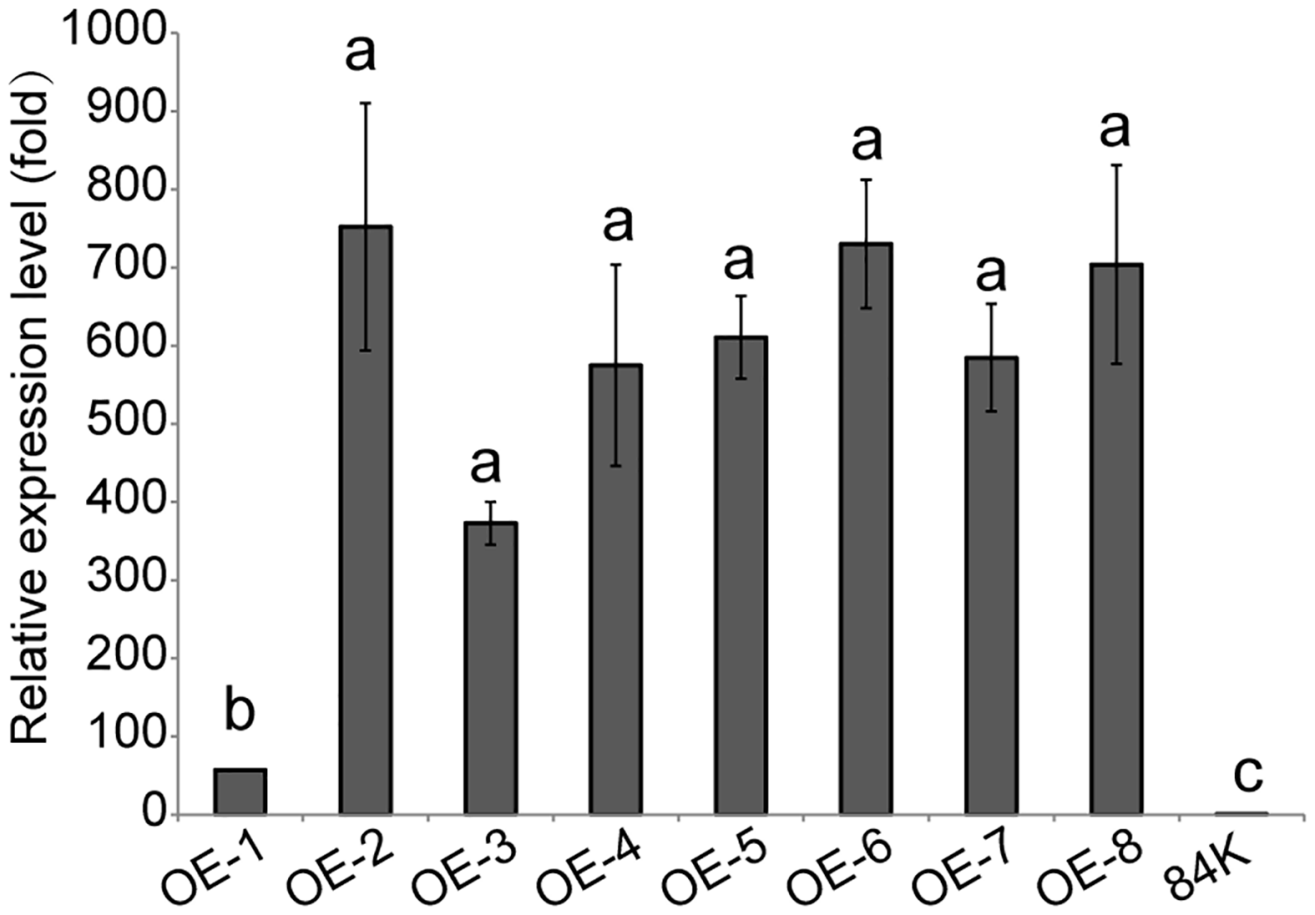
Expression of *PagSAMT* in eight lines of transgenic poplars. OE-1 to OE-8 indicates transgenic poplars containing overexpressed *PagSAMT*. 84K indicates the wild-type poplar with normal *SAMT* expression. RT-qPCR was performed with *PagSAMT*-specific primers. When the tissue culture seedings of each line grew to 10 cm tall, RNA was extracted from the middle of leaves. Expression values were normalized to the expression levels of the 84K poplar *β-Tubulin* gene in respective lines. The level of *PagSAM* expression in the 84K poplar was arbitrarily set at 1.0. Each bar represents the mean relative expression level of three independent experiments with the standard errors. Statistical differences in the means are indicated with different letters, *p < 0.05*.

The *PagSAMT* gene under the 35S promoter was transformed into 84K poplar, generating 8 independent transgenic lines, OE-1 ~ OE-8. According to RT-qPCR analysis, there were significant differences in *PagSAMT* gene expressions between the control and transgenic lines. So we selected one *SAMT* overexpressing line OE-2, which had the highest expression level of *SAMT* for the following tests (**Figure 2**). We did not observe any differences in morphological phenotype between wild-type and *SAMT*-overexpressing plants.

### Altered *PagSAMT* expression affects SA and MeSA accumulation

**Figure 3:**
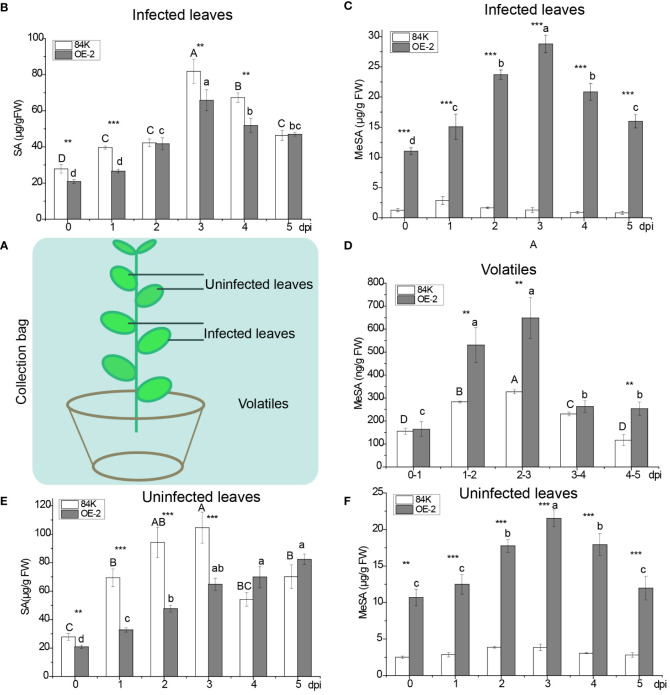
Quantification of MeSA and SA in infected and noninfected sites during pathogen inoculation. **(A)** Schematic diagram of infected and noninfected leaves of poplar. **(B)** SA levels in OE-2 and wild-type plants in infected leaves. **(C)** MeSA levels in OE-2 and wild-type plants in infected leaves. **(D)** Emission of MeSA from plants. **(E)** SA levels in OE-2 and wild-type plants in uninfected leaves. **(F)** MeSA levels in OE-2 and wild-type plants in uninfected leaves. Mean values from three independent plants are given. dpi, days past inoculation. Each bar represents the mean relative expression level of three independent experiments with the standard errors. The bars with asterisks indicate significant differences between the 84K and OE-2 lines at the same time (** *p<0.01*; *** *p<0.001*). The uppercase letters are the statistical differences of measured data with respect to the time series of the 84K line, *p<0.05*. The lowercase letters are the statistical differences of measured data during the time series of the OE-2 line, *p<0.05*.

To understand the effect of *SAMT* overexpression in poplar on SA and MeSA, we measured the content of SA and MeSA in infected and uninfected leaves and the volatilization of MeSA after inoculation with the virulent fungal pathogen *B. dothidea* (**Figure 3A**).

At the infected site, the variation of SA content in over-expressed *PagSAMT* plant tissues (OE-2) was consistent with that of the wild type (84K). SA content rose at first and then fell, and peaked on the third day. After inoculation, the content of SA in OE-2 was only significantly lower than that of wild type SA on days 0, 1, 3, and 4 (**Figure 3B**). The content of MeSA in OE-2 was significantly higher than that in the wild type during inoculation period (**Figure 3C**). The variation of MeSA content was consistent with SA content in infected leaves of OE-2 (**Figures 3B, C**). MeSA rose first and then fell, and peaked on the third day in OE-2. However, there was no significant difference of MeSA content across the infection days in 84K (**Figure 3C**). Only a small amount of endogenous MeSA evaporated into the air, and gaseous MeSA in OE-2 was significantly higher than that of the wild type on day 2, 3, and 5 after inoculation (**Figure 3D**).

At the uninfected sites, SA content in OE-2 plants increased gradually during the inoculation period. SA content in OE-2 in the early inoculation period was significantly lower than in the wild type, while in the later period, it was higher than in the wild type. The content of MeSA in transgenic poplars showed the same trend in infected and uninfected sites, but MeSA content in uninfected tissues was lower than that of infected leaves during the inoculation period (**Figures 3C**, **F**). Compared with wild-type poplar, *SAMT* overexpression in the OE-2 lines was associated with decreased SA content and increased MeSA content.

### *PagSAMT*-overexpression increases disease susceptibility to *B. dothidea*

**Figure 4:**
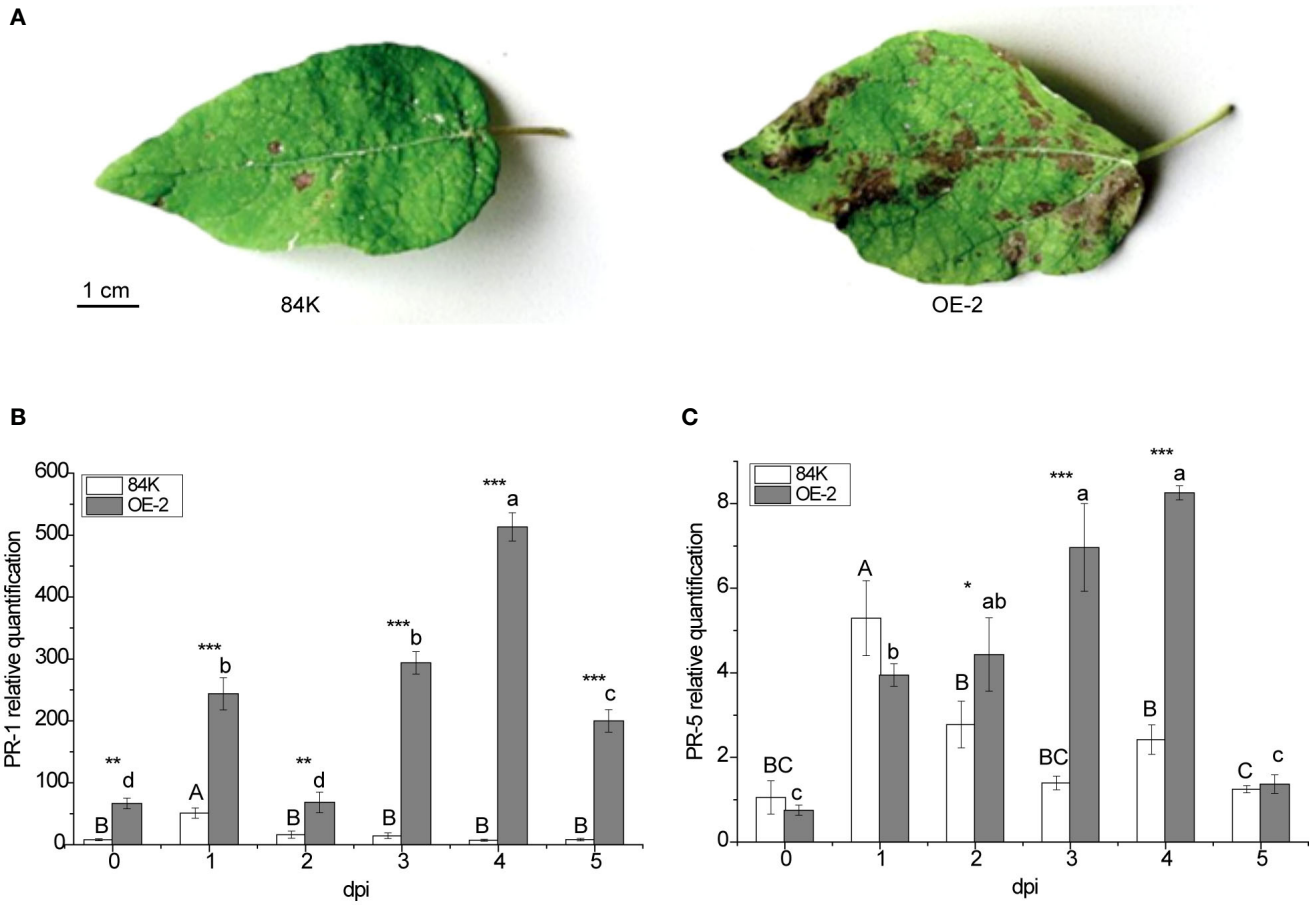
Enhanced disease susceptibility of *PagSAMT*-overexpressing plants to the virulent fungal pathogen *B dothidea* and disease-related factor responses. **(A)** Phenotype after inoculation. **(B)** Expression of *PR-1* in infected leaves. **(C)** Expression of *PR-5* in infected leaves. Mean values from three independent plants are given. dpi, days past inoculation. Each bar represents the mean relative expression level of three independent experiments with the standard errors. The bars with asterisks indicate significant differences between the 84K and OE-2 lines at the same time (* *p<0.05*; ** *p<0.01*; *** *p<0.001*). The uppercase letters are the statistical differences of measured data during the time series of the 84K line, *p<0.05*. The lowercase letters are the statistical differences of measured data during the time series of the OE-2 line, *p<0.05*.

*PagSAMT* transgenic and wild-type plants were inoculated with the virulent fungal pathogen *B. dothidea*. More severe symptoms were observed in transgenic poplar leaves 5 days after inoculation (**Figure 4A**), suggesting that overexpression of *PagSAMT* made the plants more susceptible to infection. To better understand the susceptibility mechanism of *PagSAMT* overexpressed plants, we also determined *PR* gene expression at the infected site after inoculation. In transgenic plants, overexpression of *SAMT* also resulted in high expression of the *PR-1* gene. In the OE-2 line, *PR-1* expression was 8 times higher than in the wild type before inoculation [66.62 versus 8.02] (**Figure 4B**). After inoculation, PR-1 was highly expressed in transgenic plants at the infection sites, which was significantly higher than that of the wild type during the entire inoculation period, and peaked at 70-fold higher than the wild type on day 4[493.07 vs. 7.36] (**Figure 4B**). In the *OE-2* line, the expression of PR-5 was lower than the wild-type before inoculation. After inoculation, the expression of *PR-5* gradually increased at the inoculation site and was significantly higher than that of the wild type after the second day and rapidly decreased by the fifth day (**Figure 4C**).

### Expression of *SAMT* and *SABP2* genes in poplar

The two genes, *SABP2* and *SAMT*, play important roles in the mutual transformation process of SA to and from MeSA, respectively. To understand the possible role of *PagSAMT* in regulating pathogen resistance, we measured the expression of these two genes in infected and uninfected leaves, and analyzed them in combination with the content of SA and MeSA.

**Figure 5:**
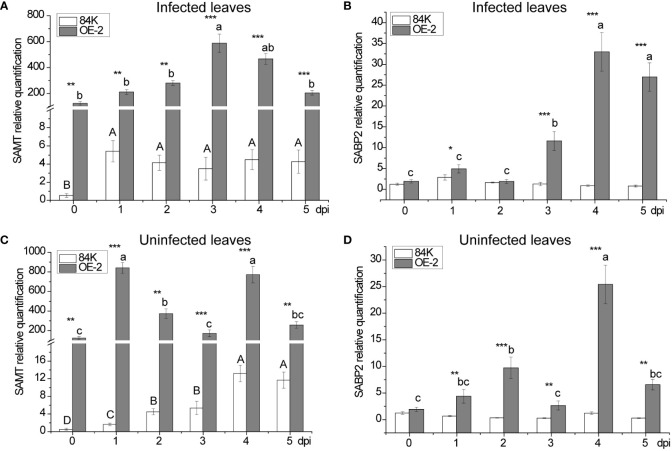
Changes in SAMT and SABP2 expression after inoculation with *B dothidea*. **(A)** Relative transcript abundance of SAMT in infected leaves. **(B)** Relative transcript abundance of SABP2 in infected leaves. **(C)** Relative transcript abundance of SAMT in uninfected leaves. **(D)** Relative transcript abundance of SABP2 in uninfected leaves. Mean values from three independent plants are given. dpi, days past inoculation. Each bar represents the mean relative expression level of three independent experiments with the standard errors. The bars with asterisks indicate significant differences between the 84K and OE-2 lines at the same time (* *p<0.05*; ** *p<0.01*; *** *p<0.001*). The uppercase letters are the statistical differences of measured data during the time series of the 84K line, *p<0.05*. The lowercase letters are the statistical differences of measured data during the time series of the OE-2 line, *p<0.05*.

*SAMT* was highly expressed in transgenic poplar, achieving 226 folds [124.60 versus 0.55] higher expression than than the wild type before inoculation (**Figures 5A, C**). After inoculation, the lowest expression level of *SAMT* in transgenic poplar inoculated tissue was 39 fold higher than the wild type (day 1) (**Figure 5A**), and the lowest expression level for non-inoculated leaves was 22 fold higher compared with the wild type (day 5) (**Figure 5C**). For SA content, expression in the wild-type at the inoculation site was 1.49 times greater than the transgenic plant (day 1) (**Figure 3B**), while expression at the non-inoculated site was 2.91 times greater than the transgenic plant (day 3) (**Figure 3E**). At the inoculated and non-inoculated sites, the highest content of MeSA in OE-2 lines was 6.58 fold (day 3) (**Figure 3C**) and 5.84 fold (day 4) (**Figure 3F**) higher than the wild type, respectively. Compared with wild-type poplar, *SAMT* overexpression in the OE-2 lines was associated with decreased SA content and increased MeSA content, but it was not obviously proportional to the expression of *SAMT*.

In the inoculated tissues of OE-2 plants, the contents of SA and MeSA also reached their maximum on the third day after inoculation when *SAMT* expression was the highest (**Figures 3B, C**). At the same time, *SABP2* began to increase significantly, but its expression level was the highest on the fourth day (**Figure 5B**), which lagged behind that *SAMT*, suggesting an increased demand for SA production to defend the OE-2 line from pathogen attack.

There were two peaks in *SAMT* expression at the non-inoculated sites of transgenic plants, which were higher than that at inoculated sites at all times except the third day when the expression was lower (**Figure 5C**). Meanwhile, the overexpression of *SAMT* also resulted in upregulation of *SABP2* expression at the non-inoculated sites, which was significantly higher than that of the wild-type, and the highest expression level of *SABP2* on day 4 was 21 times higher than that of the wild-type [25.42 versus 1.21] (**Figure 5D**).

## Discussion

Expression of *SAMT* in transgenic plants was very high, exhibiting a 226-fold increase compared with the wild type before inoculation. After inoculation, the lowest *SAMT* expression in pathogen-inoculated tissue was only 39-fold higher than the wild type. In uninfected leaves, 22- and 511-fold increases of *SAMT* expression were observed. Therefore, we predicted that *SAMT* overexpression plants might convert all SA to MeSA, however before and after inoculation, the highest levels of volatile and nonvolatile MeSA were only 6.6 times as high as the wild-type plants (day 3). Studies have shown that when *SAMT* is overexpressed in tomatoes, prior to inoculation the volatile content of MeSA in mature fruits is much higher than the expression of *SAMT*, and the content of MeSA in the inoculation site after inoculation significantly differs from the wild type (Tieman et al., 2010). In addition, studies have shown that after transforming the rice *SAMT* gene *OsBSMT1* into *Arabidopsis thaliana*, the transgenic plants convert almost all SA produced at the inoculation site into MeSA (Koo et al., 2007) and over 98% of the MeSA was evaporated into the air (Attaran et al., 2009). The proportion of gaseous MeSA produced by poplar inoculation was much lower than that of *Arabidopsis thaliana*. Different from *Arabidopsis*, when the disease-causing bacterial pathogen, *Xanthomonas campestris pv. vesicatoria* (Xcv), was used to stimulate wild-type and SAMT overexpressed transgenic tomato plants, it was found that the content of both MeSA and SA in transgenic plants significantly increased. After inoculation with Xcv, while levels of SA and MeSA were still very high, all leaves of transgenic and wild-type plants eventually showed necrosis, and transgenic plants experienced only slightly delayed symptom development. The authors suggested that overexpression of *SAMT* and the subsequent increase in MeSA synthesis disrupts normal SA metabolism, and Xcv infection aggravates these differences (Tieman et al., 2010). Compared with the over-expression of *SAMT* in *Arabidopsis* and tomato, the soybean *SAMT* gene (GmSAMT1) plays a role in soybean defense against the soybean cyst nematode (*Heterodera glycines* Ichinohe, SCN), and over-expression of *GmSAMT1* can enhance resistance to SCN (Lin et al., 2013). It was suggested that the function of *SAMT* in woody plants may be different from that in herbaceous plants. Regardless, all of the above mentioned studies indicate that *SAMT* is involved in plant defense responses against pathogens.

*BAMT*, *AMT*, and *BSMT*, which have different names based on the different substrates they act on, belong to the SABATH family. For example, SA is the substrate used by *SAMT*, benzoic acid is used by *BAMT*, SA and benzoic acid are used as substrates by *BSMT*, jasmonic acid is used as substrate by *JMT*, indole-3-acetic is used by *IAMT*, and gibberellic acid is used by *GAMT* (Seo et al., 2001; Negre et al., 2003; Zubieta et al., 2003; Qin et al., 2005; Varbanova et al., 2007). So SABATH is a large family of different genes in different species that share similar protein sequences. Some of the *BSMT*, *AMT*, *XMT* and *SAMT* sequences clustered together and the *PagSAMT* used in this study clustered with *CbSAMT* at a good confidence level (73), indicating that *PagSAMT* is a *SAMT* gene in poplar for converting SA to MeSA (**Figure 1**). However, most genes in the SABATH family have similar protein structures of close evolutionary distance and similar functions with different substrates like SA, benzoic acid, jasmonic acid, gibberellic acid or indole-3-acetic. So to confirm the function of *PagSAMT* was specific to SA, these substrates and their methylated derivatives need to be quantified in the transgenic lines for further clarification.

*PR-1*, a marker gene for SAR, was up-regulated after pathogen inoculation, but over expression did not make plants more resistant to disease. This gene could not be used as a marker for SAR in poplar plants. In our experiments, the resistance and susceptibility of poplar plants were more closely related to SA content. OE-2 plants also experienced upregulated expression of the *PR-1* gene and a large number of SAMT genes, but were more susceptible to *B. dothidea* at the end of the inoculation period. It is generally believed that the higher *PR-1* expression is, the more resistant plants are to diseases (Koo et al., 2007), but this conclusion is not applicable to all plants, and perhaps is more closely related to the species of pathogenic bacteria. Transgenic plants expressing PR-1 showed no significant resistance to tobacco Mosaic virus (Cutt et al., 1989) or alfalfa mosaic virus (Linthorst et al., 1989), but transgenic tobacco plants transformed with the *PR-1* gene of pepper experienced improved resistance to the oomycete pathogen *Phytophthora nicotianae*, and the bacterial pathogens *Ralstonia solanacearum* and *Pseudomonas syringae pv. Tabaci* (Sarowar et al., 2005). PR-1a overexpressed tobacco lines showed tolerance to two oomycete pathogens *Peronospora tabacina* and *Phytophdrip parasitica* var.*nicotianae* (Alexander et al., 1993). That *PR-1* over-expression in some plants does not increase resistance to some pathogens does not mean that the *PR-1* has nothing to do with plant resistance to pathogens in general. Before invasion of the pathogen into plant tissues, extracellular defense-related proteins had been established at the point of pathogen invasion, serving as a first line of defense. However, the accumulation of defense proteins takes time, so during this period, pathogens may invade distant tissues, and the accumulation of defense proteins serve as obstacles for subsequent invasion and thus improve SAR (Loon et al., 2006). Increased expression of *PR-1* in transgenic poplar trees before inoculation was caused by the overexpression of SAMT. The overexpression of SAMT in poplar destroyed the initial expression of *PR-1* in leaves, thereby reducing PR-1 associated disease resistance. However, we could not conclude whether OE-*SAMT* plants may be resistant to other pathogens, a factor we will evaluate resistance in subsequent experiments. *SAMT* overexpressing soybean can delay the onset of and alleviate symptoms of soybean cyst nematode inoculation (Lin et al., 2013). Analysis of SA-hyperaccumulating transgenic poplar lines showed increased jasmonate levels, however, expression of *PR* genes, frequently used as markers for SA signaling, were not correlated with SA content, but rather activated in proportion to pathogen infection (Ullah et al., 2022). So while *PR* genes could not be used as defense marker genes in poplar, SA was still involved in the resistance response.

Previous studies have shown that, in response to pathogen invasion, SA accumulates significantly at the infection site in plants (Malamy et al., 1990; Métraux et al., 1990; Rasmussen et al., 1991) and a portion of it is converted into MeSA by *SAMT*. Some of the MeSA is transported to uninfected plant parts through the phloem, while the other portion is volatilized into the air (Shulaev et al., 1997; Koo et al., 2007; Tieman et al., 2010). Once reaching the target uninfected plant parts, MeSA is transformed back into SA by SABP2, so as to prevent further infection of uninfected parts and obtain systemic resistance (Park et al., 2007; Vlot et al., 2008). After inoculation with pathogens, a large amount of SA generated at the inoculation site could not be transported by itself over a long distance (Vernooij et al., 1994), and a large amount of SA accumulation was toxic to the plants (Raskin et al., 1989; Dong et al., 2015). It may be necessary to convert SA into MeSA to alleviate the toxic effect of excessive SA on plant growth and development (Fukami et al., 2002); on the other hand, some MeSA volatilized into air is converted into SA again at the non-inoculated site (Shulaev et al., 1997), acting as a mobile signal substance for SAR (Park et al., 2007). In our study, the rise of SA at the site of OE-2 infection was correlated with the expression of *SAMT* and MeSA, while the expression of *SABP2* lagged behind. Therefore, we speculate that when plants are infected by pathogens, the SA generated at the infection site and the MeSA transformed from it *in vivo* may be regulated by *SAMT* and *SABP2*, so as to maintain the dynamic balance of SA and MeSA content in the host, which can moderately affect expression of PRs, making the plant more resistant to diseases.

However, different transgenic *SMAT* overexpression lines had different gene expression levels. OE-2 had the highest while OE-1 had the lowest relative expression level of *SMAT* compared to the control line, 84K (**Figure 2**). These differences in expression levels might be caused by insert loci or the copy number of transgenic genes (Hobbs et al., 1993; Bradford et al., 2005; Guttikonda et al., 2016). It was clearly demonstrated that multiple copies of gene inserts at the same or different loci were associated with reduced enzyme activities in tobacco (Hobbs et al., 1993), while, the copy number of transgenes could affect plant height, intermodal length, and days to maturity (Rahman et al., 2012). In this study, 8 transgenic lines were constructed and 7 had high expression levels of the target gene SMAT. So we chose only one transgenic line OE-2, for experiments to elucidate the function of SAMT in the balance between SA and MeSA in poplar in response to pathogen invasion. However, we are not sure whether the gene copy numbers and insert loci could affect the gene function or chemical contents of poplar in transgenic lines though there was no significant difference in morphological phenotype between wild-type and *SAMT*-overexpressing plants. using only one transgenic line in this study, we could not elucidate the function of *SAMT* sufficiently, thus experimentation in additional transgenic lines is needed.

*SAMT* overexpression reduced the SA content, and increased MeSA content significantly. Although the expression of *SABP2* was also up-regulated compared with the control, the difference was much smaller than that of *SAMT*. Overexpression of *SAMT* broke the balance of SA and MeSA *in vivo* and also disrupted the expression levels of *PR* genes, resulting in disordered PR genes expression. *PR-1*, in particular, was highly expressed in transgenic plants, but these plants were more susceptible to disease at the end of the inoculation period. Additionally, the expression of *PR-1* as a marker for SAR may not be appropriate in woody plants, and the relationship between SAR and SA may be closer in them. SA was transformed into MeSA with much higher expression of SAMT in OE-2, and the higher expression of *SABP2* could not make up for the reduced SA, which may account for the increased susceptibility of OE-2 to *B. dothidea.*

## Conclusions

Our study found that after inoculation with *B. dothidea*, the *SAMT* overexpressed poplar line, OE-2, could increase MeSA content massively and reduce SA content in both infected and non-infected leaves compared to the wild-type. In addition, OE-2 experienced higher expression levels of pathogenesis-related genes *PR-1* and *PR-5* but were more susceptible to *B. dothidea*, suggesting that *PR* genes were not responsible for the resistance of poplar but were associated with the SA content. Our study contributes to the research of joint regulation of *SAMT* and *SABP2* with regards to the balance between SA and MeSA in poplar to resist pathogen invasion.

## Data availability statement

The datasets generated for this study can be found on the NCBI accession Number KT429670 of full-length open reading frame of poplar 84K SAMT gene.

## Author contributions

This study was designed by XZ, YL, HD, SM and WZ. This manuscript was prepared by HD, WZ and YL. The chemical content and gene expression were measured by HD, YF and DL. The PagSAMT overexpression lines were constructed by HD, ZL and XJW. All authors contributed to the article and approved the submitted version.

## Funding

The experiment was funded by The National Key Research and Development Program of China (2017YFD0600102) and the research initiation project of HD (5101049170175).

## Conflict of interest

The authors declare that the research was conducted in the absence of any commercial or financial relationships that could be construed as a potential conflict of interest.

## Publisher’s note

All claims expressed in this article are solely those of the authors and do not necessarily represent those of their affiliated organizations, or those of the publisher, the editors and the reviewers. Any product that may be evaluated in this article, or claim that may be made by its manufacturer, is not guaranteed or endorsed by the publisher.

## References

[B1] Alexander D. Goodman R. M. Gut-Rella M. Glascock C. Weymann K. Friedrich L.. (1993). Increased tolerance to two oomycete pathogens in transgenic tobacco expressing pathogenesis-related protein 1a. Proc. Natl. Acad. Sci. United. States America 90, 7327–7331. doi: 10.1073/pnas.90.15.7327 PMC47130 8346252

[B2] Attaran E. Zeier T. E. Griebel T. Zeier J. (2009). Methyl salicylate production and jasmonate signaling are not essential for systemic acquired resistance in *Arabidopsis* . Plant Celld. 21, 954–971. doi: 10.1105/tpc.108.063164 PMC2671706 19329558

[B3] Bradford K. J. Van Deynze A. Gutterson N. Parrott W. Strauss S. H. (2005). Regulating transgenic crops sensibly: Lessons from plant breeding, biotechnology and genomics. Nat. Biotechnol. 23, 439–444. doi: 10.1038/nbt1084 15815671

[B4] Cutt J. R. Harpster M. H. Dixon D. C. Carr J. P. Dunsmuir P. Klessig D. F. (1989). Disease response to tobacco mosaic virus in transgenic tobacco plants that constitutively express the pathogenesis-related PR1b gene. Virology 173, 89–97. doi: 10.1016/0042-6822(89)90224-9 2815592

[B5] Dempsey D. M. A. Shah J. Klessig D. F. (1999). Salicylic acid and disease resistance in plants. Crit. Rev. Plant Sci. 18, 547–575. doi: 10.1080/07352689991309397

[B6] Dong X. (2001). Genetic dissection of systemic acquired resistance. Curr. Opin. Plant Biol. 4, 309–314. doi: 10.1016/S1369-5266(00)00178-3 11418340

[B7] Dong H. Duan X. Chang Z. (2015). Effect of exogenous salicylic acid on salt tolerance in perennial ryegrass. J. Beijing Forestry. Univ. 37, 128–135. doi: 10.13332/j.cnki.jbfu.2015.02.001

[B8] Du H. Klessig D. F. (1997). Identification of a soluble, high-affinity salicylic acid-binding protein in tobacco. Plant Physiol. 113, 1319–1327. doi: 10.1104/pp.113.4.1319 12223676 PMC158255

[B9] Fukami H. Asakura T. Hirano H. Abe K. Shimomura K. Yamakawa T. (2002). Salicylic acid carboxyl methyltransferase induced in hairy root cultures of *Atropa belladonna* after treatment with exogeneously added salicylic acid. Plant Cell Physiol. 43, 1054–1058. doi: 10.1093/pcp/pcf119 12354924

[B10] Green S. B. Salkind N. J. (2010). Using SPSS for Windows and Macintosh: Analyzing and Understanding Data. Prentice Hall Press

[B11] Guttikonda S. K. Marri P. Mammadov J. Ye L. Soe K. Richey K.. (2016). Molecular characterization of transgenic events using next generation sequencing approach. PloS One 11, e0149515. doi: 10.1371/journal.pone.0149515 26908260 PMC4764375

[B12] Heilman P. E. (1999). “Planted forests: poplars,” in Planted forests: Contributions to the quest for sustainable societies. Eds. Boyle J. R. Winjum J. K. Kavanagh K. Jensen E. C. (Dordrecht: Springer Netherlands), 89–93.

[B13] Hobbs S. L. A. Warkentin T. D. DeLong C. M. O. (1993). Transgene copy number can be positively or negatively associated with transgene expression. Plant Mol. Biol. 21, 17–26. doi: 10.1007/BF00039614 7678759

[B14] Hu J. Zhu W. (1997). Chitinase and n-acetyl-β-D-Glucosaminidase induced by *Dothiorella gregaria* in poplars. Acta Phytopathol. Sin. 2:1–9.

[B15] Koo Y. J. Kim M. A. Kim E. H. Song J. T. Jung C. Moon J.-K.. (2007). Overexpression of salicylic acid carboxyl methyltransferase reduces salicylic acid-mediated pathogen resistance in *Arabidopsis thaliana* . Plant Mol. Biol. 64, 1–15. doi: 10.1007/s11103-006-9123-x 17364223

[B16] Kumar D. Klessig D. F. (2003). High-affinity salicylic acid-binding protein 2 is required for plant innate immunity and has salicylic acid-stimulated lipase activity. Proc. Natl. Acad. Sci. 100, 16101–16106. doi: 10.1073/pnas.0307162100 14673096 PMC307699

[B17] Liang J. Jiang J. Liu H. Jia X. Zhao J. Wang Y. (2005). Study on the pathology of poplar-canker pathogen interaction in China. For. Res. 18, 214–221.

[B18] Liao W. Ji L. Wang J. Chen Z. Ye M. Ma H.. (2014). Identification of glutathione s-transferase genes responding to pathogen infestation in populus tomentosa. Funct. Integr. Genomics 14, 517–529. doi: 10.1007/s10142-014-0379-y 24870810

[B19] Lin J. Mazarei M. Zhao N. Zhu J. J. Zhuang X. Liu W.. (2013). Overexpression of a soybean salicylic acid methyltransferase gene confers resistance to soybean cyst nematode. Plant Biotechnol. J. 11, 1135–1145. doi: 10.1111/pbi.12108 24034273

[B20] Linthorst H. J. Meuwissen R. L. Kauffmann S. Bol J. F. (1989). Constitutive expression of pathogenesis-related proteins PR-1, GRP, and PR-s in tobacco has no effect on virus infection. Plant Cell 1, 285–291. doi: 10.1105/tpc.1.3.285 2535503 PMC159761

[B21] Li Y. Zhang W. Dong H. Liu Z. Ma J. Zhang X. (2018). Salicylic acid in *Populus tomentosa* is a remote signalling molecule induced by *Botryosphaeria dothidea* infection. Sci. Rep. 8, 14059. doi: 10.1038/s41598-018-32204-9 30232461 PMC6145909

[B22] Loon L. C. Rep M. Pieterse C. M. J. (2006). Significance of inducible defense-related proteins in infected plants. Annu. Rev. Phytopathol. 44, 135–162. doi: 10.1146/annurev.phyto.44.070505.143425 16602946

[B23] Malamy J. Carr J. P. Klessig D. F. Raskin I. (1990). Salicylic acid: A likely endogenous signal in the resistance response of *Tobacco* to viral infection. Science 250, 1002–1004. doi: 10.1126/science.250.4983.1002 17746925

[B24] Métraux J. P. Signer H. Ryals J. Ward E. Wyss-Benz M. Gaudin J.. (1990). Increase in salicylic acid at the onset of systemic acquired resistance in cucumber. Science 250, 1004–1006. doi: 10.1126/science.250.4983.1004 17746926

[B25] Molinari S. Fanelli E. Leonetti P. (2014). Expression of tomato salicylic acid (SA)-responsive pathogenesis-related genes in mi-1-mediated and SA-induced resistance to root-knot nematodes. Mol. Plant Pathol. 15, 255–264. doi: 10.1111/mpp.12085 24118790 PMC6638815

[B26] Moore J. W. Loake G. J. Spoel S. H. (2011). Transcription dynamics in plant immunity. Plant Cell 23, 2809–2820. doi: 10.1105/tpc.111.087346 21841124 PMC3180793

[B27] Morse A. M. Tschaplinski T. J. Dervinis C. Pijut P. M. Schmelz E. A. Day W.. (2007). Salicylate and catechol levels are maintained in nahG transgenic poplar. Phytochemistry 68, 2043–2052. doi: 10.1016/j.phytochem.2007.05.014 17599371

[B28] Murfitt L. M. Kolosova N. Mann C. J. Dudareva N. (2000). Purification and characterization of s-adenosyl-l-methionine: Benzoic acid carboxyl methyltransferase, the enzyme responsible for biosynthesis of the volatile ester methyl benzoate in flowers of *Antirrhinum majus* . Arch. Biochem. Biophys. 382, 145–151. doi: 10.1006/abbi.2000.2008 11051108

[B29] Negre F. Kish C. M. Boatright J. Underwood B. Shibuya K. Wagner C.. (2003). Regulation of methylbenzoate emission after pollination in snapdragon and petunia flowers. Plant Cell 15, 2992–3006. doi: 10.1105/tpc.016766 14630969 PMC282847

[B30] Park S.-W. Kaimoyo E. Kumar D. Mosher S. Klessig D. F. (2007). Methyl salicylate is a critical mobile signal for plant systemic acquired resistance. Science 318, 113–116. doi: 10.1126/science.1147113 17916738

[B31] Qin G. Gu H. Zhao Y. Ma Z. Shi G. Yang Y.. (2005). An indole-3-acetic acid carboxyl methyltransferase regulates *Arabidopsis* leaf development. Plant Cell 17, 2693–2704. doi: 10.1105/tpc.105.034959 16169896 PMC1242266

[B32] Rahman M. Rao A. Q. Batool F. Azam S. Shahid A. A. Husnain T. (2012). Transgene copy number and phenotypic variations in transgenic basmati rice. J. Anim. Plant Sci. 22, 1004–1013. doi: 10.1.1.1064.355

[B33] Raskin I. Turner I. M. Melander W. R. (1989). Regulation of heat production in the inflorescences of an *Arum lily* by endogenous salicylic acid. Proc. Natl. Acad. Sci. 86, 2214–2218. doi: 10.1073/pnas.86.7.2214 16594020 PMC286882

[B34] Rasmussen J. B. Hammerschmidt R. Zook M. N. (1991). Systemic induction of salicylic acid accumulation in cucumber after inoculation with *Pseudomonas syringae* pv *syringae* . Plant Physiol. 97, 1342–1347. doi: 10.1104/pp.97.4.1342 16668554 PMC1081169

[B35] Ross J. R. Nam K. H. D'Auria J. C. Pichersky E. (1999). S-Adenosyl-l-Methionine:Salicylic acid carboxyl methyltransferase, an enzyme involved in floral scent production and plant defense, represents a new class of plant methyltransferases. Arch. Biochem. Biophys. 367, 9–16. doi: 10.1006/abbi.1999.1255 10375393

[B36] Ryals J. A. Neuenschwander U. H. Willits M. G. Molina A. Steiner H. Y. Hunt M. D. (1996). Systemic acquired resistance. Plant Cell 8, 1809–1819. doi: 10.2307/3870231 12239363 PMC161316

[B37] Sarowar S. Kim Y. J. Kim E. N. Kim K. D. Hwang B. K. Islam R.. (2005). Overexpression of a pepper basic pathogenesis-related protein 1 gene in tobacco plants enhances resistance to heavy metal and pathogen stresses. Plant Cell Rep. 24, 216–224. doi: 10.1007/s00299-005-0928-x 15719238

[B38] Seo H. S. Song J. T. Cheong J.-J. Lee Y.-H. Lee Y.-W. Hwang I.. (2001). Jasmonic acid carboxyl methyltransferase: A key enzyme for jasmonate-regulated plant responses. Proc. Natl. Acad. Sci. 98, 4788–4793. doi: 10.1073/pnas.081557298 11287667 PMC31912

[B39] Shulaev V. Silverman P. Raskin I. (1997). Airborne signalling by methyl salicylate in plant pathogen resistance. Nature 385, 718–721. doi: 10.1038/385718a0

[B40] Tieman D. Zeigler M. Schmelz E. Taylor M. G. Rushing S. Jones J. B.. (2010). Functional analysis of a tomato salicylic acid methyl transferase and its role in synthesis of the flavor volatile methyl salicylate. Plant J. 62, 113–123. doi: 10.1111/j.1365-313X.2010.04128.x 20070566

[B41] Uknes S. Mauch-Mani B. Moyer M. Potter S. Williams S. Dincher S.. (1992). Acquired resistance in *Arabidopsis* . Plant Cell 4, 645–656. doi: 10.1105/tpc.4.6.645 1392589 PMC160161

[B42] Ullah C. Schmidt A. Reichelt M. Tsai C.-J. Gershenzon J. (2022). Lack of antagonism between salicylic acid and jasmonate signalling pathways in poplar. New Phytol. 235, 701–717. doi: 10.1111/nph.18148 35489087

[B43] Ullah C. Tsai C.-J. Unsicker S. B. Xue L. Reichelt M. Gershenzon J.. (2019). Salicylic acid activates poplar defense against the biotrophic rust fungus melampsora larici-populina *via* increased biosynthesis of catechin and proanthocyanidins. New Phytol. 221, 960–975. doi: 10.1111/nph.15396 30168132 PMC6585937

[B44] Varbanova M. Yamaguchi S. Yang Y. McKelvey K. Hanada A. Borochov R.. (2007). Methylation of gibberellins by arabidopsis GAMT1 and GAMT2. Plant Cell 19, 32–45. doi: 10.1105/tpc.106.044602 17220201 PMC1820973

[B45] Verberne M. C. Brouwer N. Delbianco F. Linthorst H. J. M. Bol J. F. Verpoorte R. (2002). Method for the extraction of the volatile compound salicylic acid from tobacco leaf material. Phytochem. Anal. 13, 45–50. doi: 10.1002/pca.615 11899606

[B46] Vernooij B. Friedrich L. Morse A. Reist R. Kolditz-Jawhar R. Ward E.. (1994). Salicylic acid is not the translocated signal responsible for inducing systemic acquired resistance but is required in signal transduction. Plant Cell 6, 959–965. doi: 10.2307/3870006 12244262 PMC160492

[B47] Vlot A. C. Klessig D. F. Park S.-W. (2008). Systemic acquired resistance: the elusive signal(s). Curr. Opin. Plant Biol. 11, 436–442. doi: 10.1016/j.pbi.2008.05.003 18614393

[B48] Wen C. Chen J. He Y. Wang F. Qian C. Wen J.. (2021). Electrophysiological and behavioral responses of red imported fire ants (Hymenoptera: Formicidae) to an essential balm and its components. Pest Manage. Sci. 77, 1971–1980. doi: 10.1002/ps.6225 33314506

[B49] Xue L.-J. Guo W. Yuan Y. Anino E. O. Nyamdari B. Wilson M. C.. (2013). Constitutively elevated salicylic acid levels alter photosynthesis and oxidative state but not growth in transgenic populus. The Plant Cell 25, 2714–2730. doi: 10.1105/tpc.113.112839 23903318 PMC3753393

[B50] Zeng D. (2002). Review of current situation of research and control on poplar diseases in China. For. Pest Dis. 21, 20–26.

[B51] Zhang W. Sun G. Xu Y. Guo B. (2009). Investigation and application of analysis methods of salicylic acid in plant tissue. Asian J. Ecotoxicol. 4, 889–897.

[B52] Zhou Y. Fu Y. Fan J. Liu Y. Gao J. Wang J.. (2007). Growth characteristics and crossability of poplar 84K. J. Northeast Forestry. Univ. 35, 11.

[B53] Zubieta C. Ross J. R. Koscheski P. Yang Y. Pichersky E. Noel J. P. (2003). Structural basis for substrate recognition in the salicylic acid carboxyl methyltransferase family. Plant Cell 15, 1704–1716. doi: 10.1105/tpc.014548 12897246 PMC167163

